# An assessment of the concentrations of pharmaceutical compounds in wastewater treatment plants on the island of Gran Canaria (Spain)

**DOI:** 10.1186/2193-1801-2-24

**Published:** 2013-01-26

**Authors:** Rayco Guedes-Alonso, Cristina Afonso-Olivares, Sarah Montesdeoca-Esponda, Zoraida Sosa-Ferrera, José Juan Santana-Rodríguez

**Affiliations:** Departamento de Química, Universidad de Las Palmas de Gran Canaria, Las Palmas de Gran Canaria, 35017 Spain

**Keywords:** Pharmaceutical compounds, Solid-phase extraction, Liquid chromatography, Mass spectrometry, Wastewater

## Abstract

An assessment of the concentrations of thirteen different therapeutic pharmaceutical compounds was conducted on water samples obtained from different wastewater treatment plants (WWTPs) using solid phase extraction and high- and ultra-high-performance liquid chromatography with mass spectrometry detection (HPLC-MS/MS and UHPLC-MS/MS), was carried out.

The target compounds included ketoprofen and naproxen (anti-inflammatories), bezafibrate (lipid-regulating), carbamazepine (anticonvulsant), metamizole (analgesic), atenolol (β-blocker), paraxanthine (stimulant), fluoxetine (antidepressant), and levofloxacin, norfloxacin, ciprofloxacin, enrofloxacin and sarafloxacin (fluoroquinolone antibiotics).

The relative standard deviations obtained in method were below 11%, while the detection and quantification limits were in the range of 0.3 – 97.4 ng·L^-1^ and 1.1 – 324.7 ng·L^-1^, respectively. The water samples were collected from two different WWTPs located on the island of Gran Canaria in Spain over a period of one year. The first WWTP (denoted as WWTP1) used conventional activated sludge for the treatment of wastewater, while the other plant (WWTP2) employed a membrane bioreactor system for wastewater treatment.

Most of the pharmaceutical compounds detected in this study during the sampling periods were found to have concentrations ranging between 0.02 and 34.81 μg·L^-1^.

## Introduction

Many modern pollution problems are a result of the intermittent or continuous release of chemical substances into the environment. Their presence is one of the main emerging issues that the organisations committed to public and environmental health have to address (Hernando et al. 2006a). Pharmaceutical compounds within this group of pollutants have raised increasing concerns over the last two decades because their effects on the environment are unknown. Thousands of tons of pharmaceuticals are used every year, in both human and veterinary medicine, and are released to the environment through metabolic excretion and improper disposal techniques. These compounds are not completely degraded at the wastewater treatment plants, and many of them are discharged into the environment through many sources and pathways (Wick et al. 2009).

These pharmaceutical compounds are objects of evaluation for their potential effects on aquatic organisms (Sanderson et al. 2004) and non-target species (Fent et al. 2006). The monitoring of these pharmaceuticals is therefore required to provide a greater knowledge with respect to their occurrence, their distribution in the environment and what effects they have on organisms when these organisms are exposed to low levels of pharmaceutical compounds (Pal et al. 2010).

The quantification of pharmaceuticals in human biological matrices such as blood, plasma or urine has been developed over a long period of time (Erny and Cifuentes 2006). Nevertheless, there is a greater difficulty in quantifying pharmaceuticals found in complex environmental samples because the concentrations of these compounds are very low and there are many compounds that can be quantified.

Gas chromatography (GC) and liquid chromatography (LC) are the most common techniques used to monitor the concentrations of organic contaminants in the environment (Hernando et al. 2006b; Zhang et al. 2011; Busetti et al. 2006; Gómez et al. 2007). Polar, non-volatile or thermally degradable compounds and their derivatives cannot be analysed by GC, and LC is an essential tool for the analysis of these types of compounds (Chen et al. 2008; Castiglioni et al. 2005). Liquid chromatography with tandem-mass spectrometry (LC-MS/MS) is the most commonly used technique (Wu et al. 2008; Baranowska and Kowalski 2010; Gros et al. 2012).

The low concentrations of pharmaceutical compounds in environmental samples render the employment of pre-treatment procedures such as the preconcentration and purification of these compounds to be necessary. The most common technique used to extract and preconcentrate pharmaceutical compounds present in environmental water samples is solid-phase extraction (SPE) (Pavlović et al. 2010; Afonso-Olivares et al. 2012; Montesdeoca-Esponda et al. 2012).

In this work, we present a monitoring of three groups of pharmaceutical compounds in wastewater samples. Group 1 consists of ketoprofen, naproxen, bezafibrate and carbamazepine, Group 2 consists of metamizole, atenolol, paraxanthine and fluoxetine and a third group consists of five fluoroquinolones, namely levofloxacin, norfloxacin, ciprofloxacin, enrofloxacin and sarafloxacin. Table 1 shows the structures and characteristics of the selected compounds. To do the monitoring, we have used the SPE, the LC-MS/MS and the UHPLC-MS/MS procedures that have previously been optimised by our group (Afonso-Olivares et al. 2012; Montesdeoca-Esponda et al. 2012). The selection of these pharmaceutical compounds was mainly based on the consumption of these compounds by the population.

**Table 1:** **List of pharmaceutical compounds, identification number, pK**_**a**_**values, chemical structure and retention times**

Group of compounds	Identification number	Compound	pK_a_	Structure	t_R_(min)
1	1	Naproxen	5.24		4.30
	2	Carbamazepine	13.9		2.14
	3	Ketoprofen	4.45		3.24
	4	Bezafibrate	-		5.52
2	5	Atenolol	9.64		7,23
	6	Metamizole	-		8,26
	7	Paraxanthine	8.5		11,23
	8	Fluoxetine	8.8		17,97
3	9	Levofloxacin	-		2,03
	10	Norfloxacin	6.4		2,23
	11	Ciprofloxacin	5.9		2,61
	12	Enrofloxacin	-		3,16
	13	Sarafloxacin	-		4,76

The water effluent samples were collected bimonthly between January 2011 and December 2011 from two different wastewater treatment plants (WWTPs) located on Gran Canaria Island in Spain. The first WWTP (denoted as WWTP1) used conventional activated sludge method for the treatment of wastewater, while the other plant (WWTP2) employed a membrane bioreactor system for wastewater treatment.

## Materials and methods

### Reagents

All the pharmaceutical compounds and fluoroquinolones used were purchased from Sigma–Aldrich (Madrid, Spain). Stock solutions containing 1000 mg·L^-1^ of each analyte were prepared by dissolving the compound in methanol, and the solutions were stored in glass-stoppered bottles at 4°C prior to use. Working aqueous standard solutions were prepared daily. Ultrapure water was provided by a Milli-Q system (Millipore, Bedford, MA, USA). HPLC-grade methanol, LC-MS methanol, and LC-MS water as well as the formic acid and the ammonium formate used to adjust the pH of the LC-MS and UHPLC-MS mobile phases were obtained from Panreac Química (Barcelona, Spain). Polyoxyethylene 10 lauryl ether (POLE) was obtained from Sigma-Aldrich (Madrid, Spain) and prepared in Milli-Q water.

### Sample collection

Water samples were collected bimonthly from the effluent of two wastewater treatment plants located in the northern part of Gran Canaria in 2011. WWTP1 utilised a conventional activated sludge treatment system, while WWTP2 employed a membrane bioreactor treatment system. The samples were collected in 2 L amber glass bottles that were rinsed beforehand with methanol and water. Samples were purified through filtration with fibreglass filters and 0.65 μm membrane filters (Millipore, Ireland). The samples were stored in the dark at 4°C and extracted within 48 hours. Influent samples were not analysed, so the degradation of the compounds during treatment was not evaluated.

### Instrumentation

The analysis of all pharmaceutical compounds except fluoroquinolones was performed in a Varian system (Varian Inc., Madrid, Spain), which consisted of a 320-MS LC/MS/MS system (triple quadrupole) equipped with an electrospray ionisation (ESI) interface, two pumps and a column valve module with an internal oven and an autosampler. The software used to control the system was MS Varian LC/MS Workstation Version 6.9.

The housing and desolvation temperatures were set at 60 and 250°C, respectively, for optimisation. Nitrogen was used as a nebuliser and a drying gas. Nebulisation was conducted at a pressure of 30 psi, and drying was conducted at a pressure of 65 psi. The capillary voltage was set to 4.5 kV in the positive mode (ESI+) and −3 kV in the negative mode (ESI–). The shield was programmed at −600/600 V (ESI+/ESI–), and the cone voltage was optimised for each compound. Collision-induced dissociation (CID) was conducted with argon as the collision gas at 1.94 psi.

The analysis of fluoroquinolones was performed in a UHPLC system from Waters (Madrid, Spain) consisting of an ACQUITY Quaternary Solvent Manager (QSM) used to load samples and wash and recondition the extraction column, an ACQUITY Binary Solvent Manager (BSM) for the elution of the analytes, a column manager, a 2777 autosampler equipped with a 25 μL syringe and a tray to hold 2 mL vials, and a ACQUITY tandem triple quadrupole (TQD) mass spectrometer with an electrospray ionization (ESI) interface. All Waters components (Madrid, Spain) were controlled using the MassLynx Mass Spectrometry Software. The electrospray ionisation parameters were fixed as follows: the capillary voltage was 3 kV, the cone voltage was 50 V, the source temperature was 120°C, the desolvation temperature was 450°C, and the desolvation gas flow rate was 800 L/hr. Nitrogen was used as the desolvation gas, and argon was employed as the collision gas.

The detailed MS/MS detection parameters for each pharmaceutical compound are presented in Table 2 and were optimised by the direct injection of a 1 mg·L^-1^ standard solution of each analyte into the detector at a flow rate of 10 μL·min^-1^.

**Table 2:** **Mass spectrometer parameters for the determination of target analytes**

Nº	Compound	Precursor ion (m/z)	Capillary voltage (Ion mode)	Quantification ion, m/z (collision potential, V)	Quantification ion, m/z (collision potential, V)
1	Naproxen	231.2	36 (ESI +)	153.1 (28.5)	170.0 (22.0)
2	Carbamazepine	237.1	40 (ESI +)	194.0 (13.5)	192.0 (17.0)
3	Ketoprofen	255.1	52 (ESI +)	209.0 (10.0)	104.9 (18.5)
4	Bezafibrate	359.8	64 (ESI -)	273.7 (15.5)	153.5 (28.5)
5	Atenolol	267.0	52 (ESI +)	145.0 (23.5)	190.0 (16.5)
6	Metamizole	218.0	30 (ESI +)	56.0 (12.5)	97.0 (11.5)
7	Paraxanthine	181.0	40 (ESI +)	124.0 (17.0)	
8	Fluoxetine	310.0	30 (ESI +)	44.0 (6.5)	148.0 (5.5)
9	Levofloxacin	362.3	40 (ESI +)	318.3 (20.0)	261.2 (30.0)
10	Norfloxacin	320.3	40 (ESI +)	302.3 (20.0)	276.2 (15.0)
11	Ciprofloxacin	332.3	40 (ESI +)	314.3 (22.0)	288.2 (18.0)
12	Enrofloxacin	360.3	40 (ESI +)	316.3 (20.0)	245.3 (25.0)
13	Sarafloxacin	386.3	40 (ESI +)	368.3 (20.0)	299.2 (30.0)

### Chromatographic conditions

For Group 1 (ketoprofen, naproxen, bezafibrate and carbamazepine), the chromatographic column used was a 2.0 mm × 50 mm, Pursuit UPS C_18_ column with a particle size of 2.4 μm. The mobile phase used was a mixture of water containing 0,2% formic acid and 5 mM ammonium formate at a pH of 2.6 and methanol. A gradient programme started the elution at a 50:50 v/v mixture of water–methanol, which changed to 40:60 (v/v) for 9 minutes, following which it returned to the initial ratio in the next minute and stayed calibrating for another minute. The flow rate was 0.2 mL·min^-1^, and the injection volume of the analyte was 10 μL.

For group 2 (metamizole, atenolol, paraxanthine and fluoxetine), the chromatographic column was a 3.0 mm × 100 mm, Sunfire™ C_18_ column with a particle size of 3.5 μm. The mobile phases, flow rate and injection volume used were the same as what was used with the analysis of group 1 compounds. The water:methanol gradient was started at 90:10 v/v. It changed to 45:55 v/v for 13 minutes and then to 35:65 in the next minute. Finally, the gradient was changed to 90:10 v/v after 16 minutes and stayed calibrating for another 5 minutes.

For the third group (levofloxacin, norfloxacin, ciprofloxacin, enrofloxacin and sarafloxacin), the analytical column was a 50 mm × 2.1 mm, ACQUITY UHPLC BEH Waters C_18_ column with a particle size of 1.7 μm (Waters Chromatography, Barcelona, Spain) operating at a temperature of 40°C. The mobile phases were water, adjusted to a pH of 2.5 with 0.1% v/v formic acid, and methanol. The analysis was performed in isocratic mode, using a 50:50 v/v water–methanol mixture at a flow rate of 0.3 mL·min^-1^. The sample volume injected was 10 μL.

### Solid-phase extraction

SPE conditions were optimised in previous studies (Afonso-Olivares et al. 2012; Montesdeoca-Esponda et al. 2012). Cartridges were conditioned with 5 ± 0.05 mL of methanol and 5 ± 0.05 mL of Milli-Q water at a flow-rate of 5 mL · min^-1^ before each run. The sample was then passed through the cartridge at a flow of 10 mL · min^-1^. A wash step was conducted using 5 ± 0.05 mL of Milli-Q water to remove any impurities. The cartridges were dried under vacuum for 10 minutes, and the analytes were eluted at an approximate flow rate of 1 mL · min^-1^.

For Groups 1 and 2, the SPE cartridge used was an OASIS HLB 6 mL/200 mg cartridge (Waters, Spain). The sample volume was 250 ± 0.15 mL at a pH of 8.00 ± 0.01 and contained 0% w/v of sodium chloride. The desorption volume was 2 ± 0.02 mL of methanol. The eluents were then evaporated under a gentle nitrogen stream and reconstituted with 1 ± 0.01 mL of LC-MS grade water. These operating conditions for SPE allowed the samples to be preconcentrated by a factor of 250.

For Group 3, the SPE cartridge used was an OASIS HLB 6 mL/200 mg cartridge (Waters, Spain). The sample volume was 200 ± 0.15 mL at a pH of 3.00 ± 0.01 and contained 0% w/v of sodium chloride, and the desorption volume was 1 ± 0.01 mL of polyoxyethylene 10 lauryl ether (POLE) (Montesdeoca-Esponda et al. 2012). These operating conditions for SPE allowed the fluoroquinolones to be preconcentrated by a factor of 200.

## Results and discussion

### Analytical parameters

An external calibration was used for the quantification of the analytes by diluting the stock solution to six concentrations ranging between 1 and 500 μg·L^-1^, where each point corresponds to the mean value obtained from three area measurements. Analysis was conducted by LC-MS/MS for Group 1 and Group 2 compounds, and UHPLC-MS/MS was used for the fluoroquinolone group. Linear calibration plots for each analyte (r^2^ > 0.99) were obtained based on their chromatographic peak areas.

The limit of detection (LOD) and the limit of quantification (LOQ) for each compound were calculated from the signal to noise ratio of each individual peak in wastewater samples spiked with the analytes. The LOD was defined as the lowest concentration that gave a signal to noise ratio that was equal to 3. The LOQ was defined to be the lowest concentration that gave a signal to noise ratio that was equal to 10. The LODs ranged from 0.3 – 7.9 ng · L^-1^ for Groups 1 and 2 and 5.3 – 11.1 ng · L^-1^ for the fluoroquinolones. The LOQs for Group 1 and Group 2 ranged from 1.1 – 26.3 ng · L^-1^, and they ranged from 17.7 to 37.0 ng · L^-1^ for the fluoroquinolones. Only Fluoxetine presented LOD and LOQ higher (97.4 and 324.7 respectively) because the transitions of fluoxetine presents more noise, so, the relation between signal and noise is lower, increasing the detection and quantification limits.

The performance and reliability of the process was studied by determining the repeatability of the quantification results for all target analytes under the described conditions. Six replicate samples were employed, obtaining relative standard deviations (RSDs) lower than 11% in all cases, indicating a good repeatability. Finally, the recoveries of the SPE methods were measured in 6 real samples and they were over 67%, except for metamizole and fluoxetine (54 and 21% respectively). Table 3 shows the analytical parameters obtained for all compounds analysed.

**Table 3:** **Analytical parameters for the SPE-LC-MS/MS and SPE-UHPLC-MS/MS methods**

Nº	Compound	RSD^a^(%) n=6	LOD^b^(ng/L)	LOQ^c^(ng/L)	Recovery (%) n=6
1	Naproxen	9.6	0.6	1.8	101.8 ± 7.0
2	Carbamazepine	10.7	0.3	1.1	105.6 ± 4.4
3	Ketoprofen	9.2	2.4	7.9	98.6 ± 9.4
4	Bezafibrate	7.8	2.9	9.6	91.6 ± 11.4
5	Atenolol	6.5	7.9	26.3	67.2 ± 4.4
6	Metamizole	7.9	6.3	21.1	54.4 ± 4.3
7	Paraxanthine	10.8	2.2	7.8	96.4 ± 10.4
8	Fluoxetine	7.7	97.4	324.7	21.0 ± 1.6
9	Levofloxacin	8.5	9.1	30.3	82.4 ± 14.0
10	Norfloxacin	8.5	8.5	28.0	85.3 ± 5.2
11	Ciprofloxacin	6.8	8.6	28.7	86.2 ± 2.1
12	Enrofloxacin	7.0	5.3	17.7	94.0 ± 6.1
13	Sarafloxacin	9.8	11.1	37.0	86.1 ± 11.2

^a^Relative Standard Derivation.

^b^Detection limits, calculated as signal to noise ratio of three times.

^c^Quantification limits, calculated as signal to noise ratio of ten times.

### Analysis of selected compounds in wastewater samples

**Figure 1:**
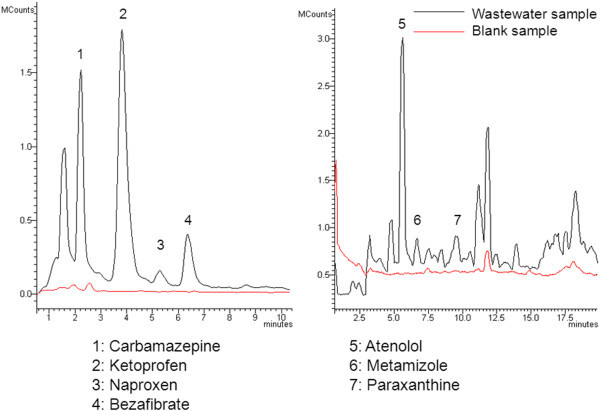
**Chromatogram of WWTP1 sample with LC/MS-MS detection for Groups 1 and 2 of compounds.**

The SPE extraction procedure was combined with the LC-MS/MS and the UHPLC-MS/MS detection methods for monitoring wastewater effluents from two different WWTPs located on Gran Canaria Island in Spain. The samples were collected once every two months over the duration of a year. The first plant (WWTP1) uses the conventional activated sludge method for the treatment of wastewater, while the second plant (WWTP2) employs a membrane bioreactor (MBR) system for wastewater treatment. Both WWTPs operate at similar daily influent sewage volumetric flow rates (500 m^3^/day for WWTP1 and 700 m^3^/day for WWTP2) and treat the wastewater from similarly sized populations (5,000 inhabitants for WWTP1 and 7,000 inhabitants for WWTP2). Figure 1, demonstrate the MRM chromatograms corresponding to wastewater samples from WWTP1 that contain compounds from Groups 1 and 2 respectively. The results of the measurements are shown in Table 4.

**Table 4:** **Concentrations in μg·L**^**-1**^**found in treated water samples from two wastewater treatment plants of Gran Canaria island**^**a**^

WWTP	Date	Naproxen	Carbamazepine	Ketoprofen	Bezafibrate	
WWTP1	Jan-2011	0.06 ± 0.01	0.51 ± 0.01	0.78 ± 0.08	2,15 ± 0.37	
	Mar-2011	0.10 ± 0.03	0.06 ± 0.01	0.22 ± 0.06	0.51 ± 0.05	
	May-2011	0.08 ± 0.01	0.02 ± 0.00	0.26 ± 0.03	1.50 ± 0.02	
	July-2011	0.25 ± 0.00	0.19 ± 0.01	1.36 ± 0.03	nd^b^	
	Sept-2011	nd^b^	nd^b^	0.07 ± 0.01	0.04 ±0.01	
	Nov-2011	0.15 ±0.01	0.04 ± 0.00	0.14 ± 0.00	0.37 ± 0.07	
WWTP2	Jan-2011	nd^b^	0.97 ± 0.03	0.11 ± 0.01	nd^b^	
	Mar-2011	nd^b^	0.36 ± 0.03	nd^b^	nd^b^	
	May-2011	0.05 ± 0.01	0.08 ± 0.00	0.30 ± 0.01	2.13 ± 0.08	
	July-2011	nd^b^	0.67 ± 0.06	0.06 ± 0.00	nd^b^	
	Sept-2011	nd^b^	0.19 ± 0.02	0.05 ± 0.01	nd^b^	
	Nov-2011	0.21 ± 0.02	0.24 ± 0.00	0.05 ± 0.00	nd^b^	
**WWTP**	**Date**	**Atenolol**	**Metamizole**	**Paraxanthine**	**Fluoxetine**	
WWTP1	Jan-2011	0.31 ± 0.01	3,45 ± 2.33	12.31 ± 0.83	nd^b^	
	Mar-2011	0.61 ± 0.20	0.41 ± 0.15	8.36 ± 0.02	nd^b^	
	May-2011	0.65 ± 0.03	nd^b^	nd^b^	nd^b^	
	July-2011	2.95 ± 0.03	0.80 ± 0.01	nd^b^	nd^b^	
	Sept-2011	0.95 ± 0.12	1.65 ± 0.21	34.81 ± 1.64	nd^b^	
	Nov-2011	0.04 ± 0,00	0.25 ± 0.08	nd	nd^b^	
WWTP2	Jan-2011	nd^b^	nd^b^	nd^b^	nd^b^	
	Mar-2011	0.07 ± 0.01	1.19 ± 1.06	nd^b^	nd^b^	
	May-2011	nd^b^	8.25 ± 0.19	nd^b^	nd^b^	
	July-2011	0.12 ±0.01	0.24 ± 0.02	nd^b^	nd^b^	
	Sept-2011	0.26 ± 0.04	0.62 ± 0.14	nd^b^	nd^b^	
	Nov-2011	0.04 ± 0.00	0.25 ± 0.08	nd^b^	nd^b^	
**WWTP**	**Date**	**Levofloxacin**	**Norfloxacin**	**Ciprofloxacin**	**Enrofloxacin**	**Sarafloxacin**
WWTP1	Jan-2011	4.40 ± 0.20	nd^b^	nd^b^	nd^b^	nd^b^
	Mar-2011	2.93 ± 0.17	nd^b^	11.1 ± 0.75	nd^b^	nd^b^
	May-2011	3.70 ± 0.28	nd^b^	20.3 ±1.81	nd^b^	nd^b^
	July-2011	nd^b^	nd^b^	nd^b^	nd^b^	nd^b^
	Sept-2011	nd^b^	nd^b^	nd^b^	nd^b^	nd^b^
	Nov-2011	0.44 ± 0.03	nd^b^	nd^b^	nd^b^	nd^b^
WWTP2	Jan-2011	5.90 ± 0,59	nd^b^	nd^b^	nd^b^	nd^b^
	Mar-2011	14.1 ± 0.92	nd^b^	nd^b^	nd^b^	nd^b^
	May-2011	6.24 ± 0.28	nd^b^	16.02 ± 0.82	nd^b^	nd^b^
	July-2011	nd^b^	nd^b^	nd^b^	nd^b^	nd^b^
	Sept-2011	nd^b^	nd^b^	nd^b^	nd^b^	nd^b^
	Nov-2011	nd^b^	nd^b^	nd^b^	nd^b^	nd^b^

^a^n = 3.

^b^nd = not detected.

We can observe that the concentrations of the group 1 compounds range consistently from 0.05 and 0.30 μg·L^-1^ for naproxen, carbamazepine and ketoprofen. Bezafibrate exhibits concentrations ranging between 0.04 and 2.15 μg·L^-1^. WWTP1 has higher naproxen, ketoprofen and bezafibrate concentrations that WWTP2 and a similar effluent concentration for carbamazepine.

There are more notable differences in the analysis of the pharmaceuticals in Group 2. In WWTP1, atenolol concentrations range between 0.04 and 0.95 μg·L^-1^, except in one sample (July 2011), where the concentration was 2.95 μg·L^-1^. Metamizole concentrations range between 0.25 and 3.45 μg·L^-1^, while the concentrations of paraxanthine are higher, ranging between 8.36 and 34.81 μg·L^-1^. The higher detected concentrations of paraxanthine can be explained through the fact that this compound is a metabolite of caffeine in the human body. In WWTP2, the concentrations of atenolol and metamizole are lower, except for metamizole in the May 2011 sample. In WWTP2, paraxanthine was not detected at all, while fluoxetine was not detected in either of the WWTPs.

The concentrations of the fluoroquinolones in both WWTPs are similar, ranging between 2.93 and 14.1 μg·L^-1^ for levofloxacin and between 11.1 and 20.3 μg·L^-1^ for ciprofloxacin. Norfloxacin, enrofloxacin and sarafloxacin were not detected in either of the WWTPs.

In summary, the concentrations of the pharmaceuticals and antibiotics detected at the wastewater treatment plant that operated with a membrane bioreactor treatment system are lower than that of the WWTP operating with a traditional technique such as activated sludge. Therefore, if the influent water quality of both the WWTPs is similar, the membrane bioreactor technique (MBR) can be said to be more efficient than the activated sludge technique.

## Conclusions

A survey on the presence of pharmaceutical compounds in two wastewater treatment plants on the island of Gran Canaria in Spain was conducted. The scope of this study included eight common pharmaceutical compounds (naproxen, carbamazepine, ketoprofen, bezafibrate, atenolol, metamizole, paraxanthine and fluoxetine) and five fluoroquinolones (levofloxacin, norfloxacin, ciprofloxacin, enrofloxacin and sarafloxacin). Wastewater effluent samples were collected bimonthly in 2011. During the monitoring period, 9 analytes were detected in all samples, with analgesics, anti-inflamatories and lipid regulators being the most frequently detected compounds.

A group of fluoroquinolones was selected for analysis because they were considered “priority pollutants” due to their potential hazardous effects on the aquatic environment.

The results show that the elimination of most of the analysed compounds is incomplete, but the membrane bioreactor technique is the more efficient of the two wastewater treatment process analysed in the removal of pharmaceutical compounds, and it results in lower effluent concentrations for most of the compounds in comparison with the activated sludge technique.

The results obtained in this monitoring work support the motivation for including pharmaceutical compounds in the monitoring of wastewater effluent quality.
